# Laryngotracheal Complications after Intubation for COVID-19: A Multicenter Study

**DOI:** 10.3390/life13051207

**Published:** 2023-05-18

**Authors:** Estefanía Hernández-García, Rosa Hernández-Sandemetrio, Ana Quintana-Sanjuás, Enrique Zapater-Latorre, Ramón González-Herranz, Lorena Sanz, Rosa Reboll, Beatriz Pallarés-Martí, Montserrat Ollé-Moliner, Paula Martínez-Pascual, Itziar Gotxi, Araly Chacón-Uribe, Guillermo Plaza

**Affiliations:** 1 Department of Otorhinolaryngology, Hospital Universitario de Fuenlabrada, Universidad Rey Juan Carlos, 28042 Madrid, Spain; fanyhdzgarcia@hotmail.com (E.H.-G.); rgherranz@salud.madrid.org (R.G.-H.); 2 Department of Otorhinolaryngology, Hospital General Universitario, 46014 Valencia, Spain; rhernandezorl@gmail.com (R.H.-S.); ezapaterlatorre@yahoo.es (E.Z.-L.); 3 Department of Otorhinolaryngology, Hospital Universitario Lucus Augusti, 27003 Lugo, Spain; anaquintanasanjuas@gmail.com; 4 Department of Otorhinolaryngology, Hospital Universitario Torrejón, 28850 Madrid, Spain; lorena_sanzlopez@yahoo.es; 5 Department of Otorhinolaryngology, Hospital Universitario Sagunto, 46115 Valencia, Spain; eboll540@yahoo.es; 6 Department of Otorhinolaryngology, Consorci Corporació Sanitaria Parc Taulí Sabadell, 08208 Sabadell, Spain; beatriz.pallares.marti@gmail.com; 7 Department of Otorhinolaryngology, Hospital Son Llàtzer, 07198 Palma de Mallorca, Spain; molle@hsll.es; 8 Department of Otorhinolaryngology, Hospital Universitario Severo Ochoa, 28914 Madrid, Spain; pmartinez.3@alumni.unav.es; 9 Department of Otorhinolaryngology, Hospital de Galdakao-Usansolo, 48960 Bizkaia, Spain; i.gotxierezuma@gmail.com; 10 Department of Otorhinolaryngology, Hospital Universitario Fundación Jiménez Diaz, 28042 Madrid, Spain; araly.chacon@quironsalud.es

**Keywords:** laryngeal complications, COVID-19, prolonged intubation, tracheostomy, subglottic stenosis

## Abstract

Many of the patients with COVID-19 have suffered respiratory distress requiring prolonged endotracheal intubation (ETI) resulting in laryngotracheal complication with an impact on breathing, phonation, and swallowing. Our aim is to describe laryngeal injuries diagnosed after ETI in patients with COVID-19 in a multicentre study. Methods: A prospective descriptive observational study was conducted from January 2021 to December 2021, including COVID-19 patients with laryngeal complications due to ETI diagnosed in several Spanish hospitals. We analyzed the epidemiological data, previous comorbidities, mean time to ICU admission and ETI, need for tracheostomy, mean time on invasive mechanical ventilation until tracheostomy or weaning, mean time in ICU, type of residual lesions, and their treatment. Results: We obtained the collaboration of nine hospitals during the months of January 2021 to December 2021. A total of 49 patients were referred. Tracheostomy was performed in 44.9%, being late in most cases (more than 7–10 days). The mean number of days of ETI until extubation was 17.63 days, and the main post-intubation symptoms were dysphonia, dyspnea, and dysphagia, in 87.8%, 34.7%, and 42.9%, respectively. The most frequent injury was altered laryngeal mobility, present in 79.6%. Statistically, there is a greater amount of stenosis after late ETI and after delayed tracheostomy, not observing the data with the immobility alterations. Conclusion: The mean number of days of ETI was long, according to the latest guidelines, with the need for several cycles of pronation. This long ETI may have had an impact on the increase of subsequent laryngeal sequelae, such as altered laryngeal mobility or stenosis.

## 1. Introduction

The coronavirus disease 2019 (COVID-19) pandemic has caused a worldwide health challenge due to its severe respiratory effects leading to an increased need for ventilatory support in intensive care units (ICU). This resulted in an increased number of endotracheal intubations (ETI), which were often prolonged prior to tracheostomy.

Therefore, the need for tracheotomies has also increased at this time, not only because of the need for respirators for the mass of patients who need ETI, but also because the type of COVID-19 patient implies other risks, one of which is prolonged ETI needing tracheotomy for weaning, in most cases [1,2,3,4,5,6,7].

The pathophysiology of severe acute respiratory syndrome coronavirus-2 (SARS-CoV-2) infection was initially unknown, and its rapid transmission caused the collapse of many hospitals and ICU in the first waves. As a result of vaccination campaigns, this pandemic seems to be more under control, but infections are still present, as well as comorbidity and deaths [1,2]. The seriousness of this disease lies in its pulmonary involvement, which can lead to severe respiratory distress requiring mechanical ventilation, endotracheal intubation, and even tracheostomy, with a mortality rate of around 2–5% [3,4,5]. In the early days of the pandemic, the high work overload and fear of contagion led to measures to minimize the risk of aerosolization, such as maintaining high tube pressures and prolonging the time of ETI until tracheostomy far beyond the recommended time (7–10 days) following the new update protocols, resulting in more than 20 days of ETI in many occasions [5,6,7,8]. There was a lack of protocols on the performance of tracheotomies during the COVID-19 pandemic. At the beginning, the initial recommendations were based on avoiding prolonged aerosolization maneuvers, and reinforcing the use of personal protective equipment, interventions carried out by expert professionals, and negative pressure rooms to minimize the risk of infection. This delay was also based on the fact that the viral load seems to be reduced with fatigue two weeks after the onset of symptoms, thus reducing the risk of aerosolization and contagion of healthcare workers during a tracheostomy.

However, as other studies have shown [9,10,11,12,13], ETI beyond seven days can cause laryngeal lesions such as edema, granulomas, ulcers in the glottic level, or impaired laryngeal mobility. Chronic effects of these injuries include airway stenosis, laryngeal stenosis below, at, or above the vocal folds, mucosal scarring and abnormalities, vocal fold vibration, vocal fold fixation, post-intubation phonatory failure, and swallowing problems. While each of these conditions can occur with varying degrees of severity, even mild disturbances of precise laryngeal function can lead to an important functional compromise with an impact on the quality of life of patients suffering from these complications.

Indeed, the mobilization of patients in pronation cycles to improve alveolar recruitment has also been frequent during the ETI of COVID-19 patients. As a consequence, the possible fragility of the laryngeal mucosa in this disease may have favored the appearance of laryngeal lesions due to prolonged intubation and pronation. Many of these complications may go unnoticed in a patient who has overcome this serious disease; however, the sequelae cannot go unnoticed by specialists as they may justify the persistence of a clinical condition related to breathing, phonation, and swallowing.

Thus, prolonged ETI and pronation, together with other individual factors, could favor the formation of lesions in the upper airway, producing motor alterations of the upper airway, stenosis, paralysis, or granulomas [9,10,11,12,13]. These airway injuries have several treatment options, all of which are very laborious and involve several specialists, depending on each hospital and access to different specialties. Its treatment varies from open resection with recanalization of the tube, to endolaryngeal treatment with different accesses and tools to reduce or eliminate the stenosis. Even with all these treatments, the success of these surgeries is limited and requires, in many or most cases, several interventions or the need to carry out different strategies on a single lesion in a single patient.

Several studies [5,6,7,8,9,10,11,12] conclude that in patients who require prolonged intubation, laryngeal lesions in different structures are inevitable in many cases, and that, to minimize them, it would be interesting to perform an early tracheotomy. In addition, tracheostomy reduces the risk of ventilator-associated pneumonia and reduce the number of days on mechanical ventilation. Moreover, tracheostomy carries other advantages in front of the prolonged endotracheal intubation as the necessity of lower sedation allows us to maintain oral communication.

COVID-19 is increasingly understood to be a systemic disease with extrapulmonary manifestations in the cardiovascular, renal, neurologic, endocrine, and gastrointestinal systems and beyond, requiring multidisciplinary management from various specialists. The role of the otolaryngologist is similarly important in the care of patients with COVID-19 and includes contributions in the management of the surgical airway. Otolaryngologists must also recognize the laryngeal complications in voice, airway, and swallowing difficulties that seem to be related to prolonged intubation in these patients and consider these etiologies in the differential diagnosis of patients presenting to the outpatient setting [10].

As a consequence, during the pandemic, many tracheostomies have been performed by otolaryngology services around the world. However, on many occasions, such a procedure has been delayed for more than 20 days after ETI, due to, as we have commented, the great care pressure in the ICUs, the fear of contagion during aerosolization maneuvers, and the respiratory instability of many patients who need to continue with pronation cycles. This has resulted in an increase in laryngeal lesions after ETI detected by ENT throughout the world. Therefore, it would be interesting to know its incidence and pathophysiology. The aim of this study was to warn of possible laryngeal complications after prolonged ETI due to COVID-19 that may go unnoticed after discharge from the ICU, with the collaboration of several hospitals in Spain.

## 2. Materials and Methods

An observational, descriptive, prospective, multicenter study was designed. To carry out the multi-institutional study, patients were recruited from all the hospitals who agreed to provide data throughout all the regions of Spain, with the acceptance of one physician per institution for subsequent writing and preparation.

The study was promoted and publicized by the Spanish Society of Otorhinolaryngology (SEORL), by means of webinars and oral paper presentations at the National Meeting in 2020 and 2021, for the nationwide collaboration of all the hospitals that wished to join the project.

The approval of each hospital’s ethics committee was obtained (IRB number: APR 21/10), including all consecutive patients with a history of ETI due to COVID-19 with symptomatology and/or pharyngolaryngeal lesions subsequently referred to the Department of Otolaryngology of each hospital. The study period was started at January 2021 and finished at December 2021.

Inclusion criteria were prolonged ETI in ICU for COVID-19 with subsequent pharyngo-laryngeal symptoms (dyspnea, dysphonia, and dysphagia) with or without pathological findings on laryngeal fibroscopy.

We analyzed the issuing hospital, age, sex, most frequent comorbidities, body mass index (BMI), comorbidities including heart failure, coronary artery disease, cerebrovascular disease, and respiratory disease (COPD or asthma), tobacco use, diagnostic test for COVID-19 infection, general symptoms, otorhinolaryngology (ENT) symptoms at diagnosis, type of intubation, number of intubation attempts, number of intubation tubes, need for pronation, number of pronation cycles, need for laryngeal fibroscopy, number of intubation days, need or not for tracheostomy, post-intubation complications, ENT symptoms after intubation, location of lesion on fibroscopy (glottis, supraglottis, or subglottis), type of lesion, whether or not the lesion was treated, type of treatment, sequence in multiple treatments, resolution, and sequelae.

The data obtained were recorded in a Microsoft Excel Version 16.72 database. Data are presented as absolute numbers, percentages, and standard deviation. In addition, the statistical relationship of prolonged ETI with stenosis or paralysis and the influence of tracheotomy in these cases were analyzed. For this, the corresponding statistical tests were performed (Pearson chi-squared test). Being a prospective study, power analysis was performed to obtain a minimum of 50 cases. A *p* value < 0.05 was considered statistically significant. All analyses were performed using SPSS software (v. 18.0, SPSS Inc., Chicago, IL, USA).

## 3. Results

We obtained the collaboration of nine hospitals distributed throughout Spain (Table 1) for 12 months, from January 2020 to December 2021. Four of them are in the Community of Madrid, two in the Community of Valencia, one in Community of Catalonia, one in Community of Balearic Islands, and one in Basque Country.

The total number of patients referred was 49 patients, 59.2% male (29 patients) and 40.8% female (20 patients) (Table 2). The mean age was 60.6 years (range 33 to 80 years). The most frequently observed comorbidities were arterial hypertension, diabetes mellitus (DM), and dyslipidemia (DL), as well as obesity, with a mean BMI of 28.9 (range 10 to 48.1). Tobacco use was observed in 13 patients (26.5%: six active and seven recent ex-smokers) (Table 2).

COVID-19 was diagnosed in most cases by polymerase chain reaction (PCR) (*n* = 47), and by antigen detection in only two patients, always obtained from a nasopharyngeal swab. The main presenting symptoms of infection were fever, cough, asthenia, myalgia, and headache (Table 3). The most frequent symptom prior to intubation were dyspnea, fever, and cough. Most intubations were ETI (97.9%, 48 patients), and only one patient received nasotracheal intubation. The mean number of intubation attempts was 1.3 (range 1 to 4). The mean tracheal tube size was 8 in men and 7 in women. Pronation cycles were required in 73.5% of patients in their ICU treatment, with a mean of 5.3 days (range 1 to 30 days). Tracheostomy was performed in 44.9% (22 patients), being late (beyond 7 days) in most cases (mean 16.42 with a range of 7–31 days). The mean number of ETI days to extubation was 17.63 days (range 4–77 days) The main post-intubation symptoms and reasons for consultation of the ENT specialist after discharge from the ICU were dysphonia, dyspnea, and dysphagia, in 87.8%, 34.7%, and 42.9%, respectively (Table 4).

Nasal flexible fibroscopy was performed in 100% of the patients to be able to show an objective damage caused after ETI, at various times: at the ICU bedside, while hospitalized, or after hospital discharge during follow-up visits.

**Figure 1:**
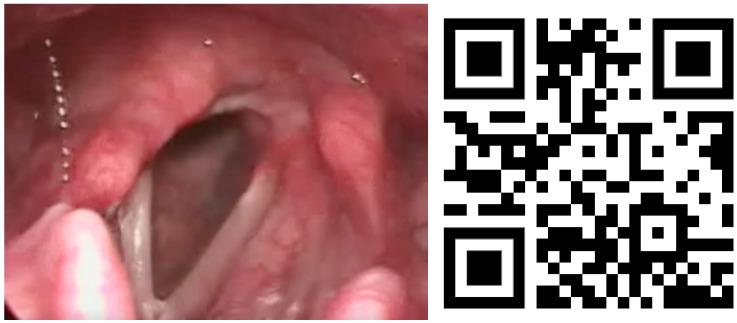
Unilateral ankylosis image and QR video. Video also available as Supplementary Material Video S1.

**Figure 2:**
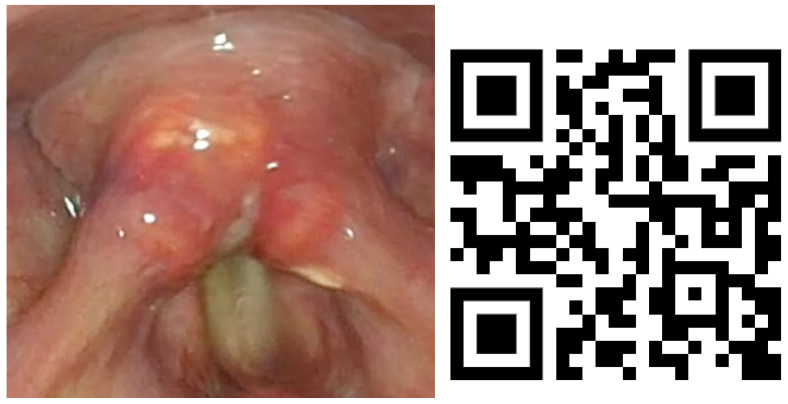
Bilateral vocal fold paralysis image and QR video. Video also available as Supplementary Material Video S2.

**Figure 3:**
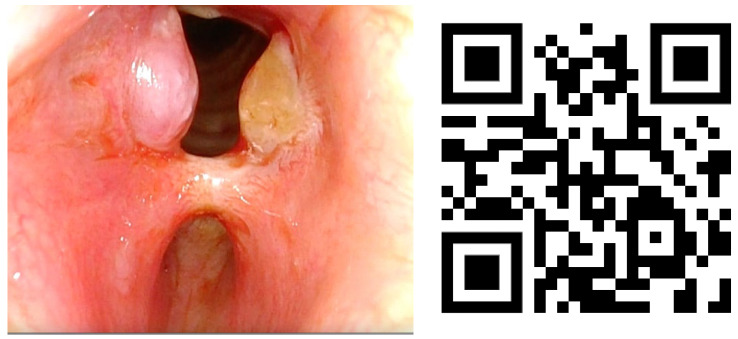
Subglottic stenoses and granulomas with QR video. Video also available as Supplementary Material Video S3.

Lesions in the glottic plane were observed in 89.8% of patients (Table 5). The most frequent lesion was altered laryngeal mobility (Figure 1 and Figure 2) in 79.6% (39 patients), followed by six subglottic stenoses (Figure 3), six granulomas, two atrophies, three synechiae, one tracheocutaneous fistula, and one glottic edema. Several of them could be observed simultaneously in the same patient.

Only 11 of these lesions were not treated, due to spontaneous improvement. The rest of the patients underwent different treatments (medical, speech therapy, or surgery), with the possibility of combining them. Surgery was necessary in 34.7% of the cases (17 patients), of which six patients underwent surgery (laser or combined surgeries) as the sole and decisive treatment, and 11 patients had chronic sequelae.

At the time of study closure, 20 patients (40.2%) had been discharged from ENT follow-up, due to their clinical recovery or disappearance of the lesions. The remaining 29 cases (59.2%) were still being followed for their sequelae after intubation or after the relevant ENT surgeries.

We found a statistical relationship between performing a delayed tracheotomy and the possibility of presenting a subglottic stenosis with a *p* = 0.043; indeed, prolonged ETI (>7 days) was related to the appearance of stenosis. Analyzing subglottic stenoses, most were associated to posterior glottic stenosis, a well-known complication of prolonged endotracheal intubation. In this series, it was not related to the tracheostomy; rather, it was related to prolonged endotracheal intubation.

As the number of cases of paralysis or alteration of laryngeal movement by mechanic etiology after prolonged ETI was small, no statistical significance was observed (*p* = 0.321), but a trend towards a greater frequency was observed in these cases.

## 4. Discussion

Unfortunately, during the COVID-19 pandemic, ETI length has had to be prolonged more than the recommended timing, until tracheostomy or weaning was performed, according to ICU clinical management guidelines in most countries [3,5,6,7,8,14,15,16]. This delay has resulted in many tracheostomies being performed more than 10 days from initial ETI.

This increase in ETI days is due, at the beginning of the pandemic in 2020, to ignorance of the disease itself, to the attempt to reduce airway manipulation to avoid aerosolization and contagious risk, and to the need for more days due to the pathophysiology of the disease due to respiratory instability of patients, among other causes. As a result, the airway is affected to a greater or lesser extent, by being in contact with an exogenous material, such as the endotracheal intubation tube, its position, and the higher pression ball and the area on which it rests, which changed in prone cycles, producing a series of laryngotracheal complications that have an effect and need assessment by various professionals, including ENTs, speech therapists, and UCI professionals.

This has augmented dyspnea, dysphonia, and dysphagia symptomatology after laryngeal and tracheal ETI with the arrival of new patients to our practice, with an increased number of lesions observed on fibroscopies performed by ENT departments across the country.

According to our study, 90% of patients with pharyngo-laryngeal symptoms after prolonged ETI (more than 7 days) had objectifiable organic lesions. Thus, the most common symptom with which the ICU doctors most often notified the otorhinolaryngologist for the evaluation of the COVID-19 patient was dyspnea and dysphonia or voice alteration after weaning. This made us think about the diagnosis of movement impairment of the vocal cord or laryngeal stenoses, needing to demonstrate it with a fibroscopic exploration at bedside, as early as possible. For this, a portable fiberscope and camera equipment are needed to be able to see the lesions in the patient’s bed. Another option is to wait for that patient to no longer be hospitalized to be able to mobilize him to the otorhinolaryngology office and be able to explore him with the fiberscopes housed in those places. The latter can delay the diagnosis of a laryngeal lesion, since the hospital stay in the ICU of a patient with COVID-19 is prolonged, even up to several months. On the other hand, there were patients who are diagnosed late, after hospital discharge, months after the COVID-19 infection.

It is known that ETI can cause laryngotracheal complications due to several mechanisms: first, an urgent and traumatic intubation may lead to disarticulation of one or both arytenoid cartilage; second, tube balloon pressure greater than that of the submucosal capillaries may develop tube decubitus that can lead to ischemic tissue damage at the mucosal level, especially at the posterior commissure. According to several authors [5,11,17,18,19,20,21,22,23], ulcers of the laryngeal mucosa led to fibrosis that can leave sequelae such as stenosis or ankylosis of the cricoarytenoid joint, requiring surgical treatment in most cases, 80% in our case, as supported by other authors [24,25,26,27].

These lesions are observed by ICU professionals due to a wide variety of symptoms, but one of them is stridor or dyspnea at weaning, or the impossibility of weaning if the stenosis is very severe. Given this, the ENT must be attentive and perform a fibroscopy while being aware of the possible lesions that can be seen. If feasible, the ideal would be for the fibroscopy to be performed by an ENT expert in laryngology assisted by another ENT, and for a recording to be made to reproduce it as many times as necessary to corroborate the diagnosis.

In our study, the most frequently detected lesion was unilateral vocal immobility, as has been described in other studies [12,27,28,29]. Vocal immobility can be caused either mechanically, as described above, or neurogenically. In this case, it is the pressure of the balloon tube at the cricothyroid joint that causes compression of the recurrent laryngeal nerve at its entry into the larynx and causes ischemic damage with restriction of vocal mobility. It should also be noted that the neurotropism of the SARS-CoV-2 virus itself can cause paralysis, as has been seen in other neuropathies (loss of smell and sudden deafness) due to this infection [30]. Prolonged intubation is described in the literature as one of the most frequent causes of mechanically caused vocal immobility [31], as in our study, and, with the prolongation of ETI and viral pathogenesis, it has been seen that this could be increasing. It should be noted that, despite not obtaining statistical significance, a trend towards a possible relationship is observed.

On the other hand, the longer the duration of ETI, the greater the risk of iatrogenesis due to laryngeal lesions [18,19]. The general recommendation is to perform a tracheostomy after 7–10 days, but, during these severe pandemic times, this period has been delayed due to the high pressure of care and in order to avoid aerosolization maneuvers. In our study, tracheostomy was delayed in 100% of cases (beyond 7 days) with an average of 16.35 days. This could be one of the hypotheses for the appearance of more lesions after intubation in this type of patient than in others intubated for another pathology in other periods outside the COVID-19 pandemic (Table 6) [5,6,7,8,9,10,11,12,13,23,32,33,34,35,36,37,38,39,40,41,42,43,44,45,46,47].

In a large prospective study on 421 patients under ETI diagnosed with COVID-19, Félix et al. [29] found that 172 (40.9%) were finally discharged. Outpatient evaluation by videoendoscopy was performed in 95 patients (55.2%) approximately 100 days after extubation. Laryngotracheal lesions were observed in 38 patients (40%), with 17.9% diagnosed with laryngotracheal stenosis or unilateral immobility while 6.3% had severe stenosis (grades 3 and 4). The factors presenting statistical significance for the development of laryngotracheal lesions were the endotracheal tube size; prone position over the ETI period; and the increased leukocyte count, D-dimer, prothrombin time (PT), and international normalized ratio (INR) on the day ETI was performed.

A recent systematic review presented by Kelly et al. [46] included six cohorts and a total number of 436 patients. Persistent features of laryngotracheal complication identified were airway abnormalities (18.9–27%), dysphonia (13.2–60%) and dysphagia (23–33%). Persistent laryngotracheal complication was associated with ICU length of stay, respiratory diagnosis, and tracheostomy. The prolonged duration of ETI over 20 days has demonstrated up to 30% of muscle mass loss, and, in combination with prone ventilation, this may be expedited. A combination of these factors may contribute to sarcopenia-related dysphagia, which has been demonstrated in elderly patients.

As reported by Almutairi et al. [40], the timing of tracheostomy among COVID-19 patients is still controversial, and several guidelines on this topic have not yet reached a consensus. Earlier recommendations encouraged postponing tracheostomy beyond 14 days, when the danger of viral transmission is minimal and the prognosis of the critically ill patient becomes clearer [47]. However, several authors and a recent systematic review and meta-analysis of non-COVID-19 patients found that tracheostomy performed early (within 7 days) was found to have a lower incidence of mortality and ventilator-associated pneumonia, as well as a shorter period of mechanical ventilation and ICU stay [48,49].

Health workers must consider that this problem could reoccur in a health emergency such as the one experienced during the COVID-19 pandemic, and this should lead to the creation of new protocols for action in these situations, especially in those that imply another wave of massive intubations such as this time, so that with them, both the risk of infection of professionals and the risk of injuries after ETI are reduced.

Instead, it has also been described that, in SARS-CoV-2 respiratory infection, the laryngeal mucosa is more fragile due to inflammatory changes, which makes it more vulnerable to the factors described, so that, in these patients, tracheostomy should be brought forward [6,7,23,34]. However, according to our study, this measure is still not being carried out, with delay due to the risk of aerosolization prevailing over that of preventing post-intubation injury.

Subglottic stenosis, laryngeal mucosa ulceration, and glottic granulomas have also been frequently observed and may be a consequence of this injury to the base mucosa, the number of days of intubation, or the size of the tube, as described in other studies [11,27,29,35,36,37,38,39,40]. The laryngotracheal complications can derivate in fibrosis with secondary arytenoid ankylosis and laryngeal movement impairment.

These types of injuries are ones that deserve special care, since their treatment is highly varied in the literature. A good diagnosis is essential, requiring a quality recording with a good camera in our fiberscope, since they are lesions that can go unnoticed when they are under the vocal cords and can be difficult to see if you do not think about them and you are not a specialized ENT in larynx. In these situations, advanced laryngeal surgical treatment is required (CO_2_ laser combined with cold endolaryngeal surgery, or blue laser surgery in office for strictures or when there is the need for a type II laryngoplasty for paralysis), often after several attempts with other non-surgical treatments (such as injection of intralesional corticosteroids or treatment with oral corticosteroids in a descending regimen).

In our study, we have indeed observed that prolonged ETI or performing a delayed tracheotomy entails a higher risk of stenosis; thus, this must be considered in the future for possible changes in the design of the joint action protocols of the ICU and ENT. Stratakos et al., published in 2022 [27], obtained a good series of subglottic stenosis after COVID-19 intubation. This article concludes that the association of prolonged intubation, repeated maneuvers in pronation cycles, and the high number of tubes used for intubation, as well as other disease-specific factors, could be involved in the formation of these strictures. They also studied the histopathology accompanying these lesions, concluding that they do not differ from other strictures analyzed in other periods outside the COVID-19 pandemic. Thus, they confirmed that careful prevention, early detection, and effective treatment of these lesions are needed. The relation between pronation and laryngeal complications after COVID-19 are currently unknown, but it may increase cuff pressures on the trachea walls due to the bending of the tube [10].

In our study, most patients were referred late (more than 30 days on average) after discharge from the ICU. We believe that referral to the ENT specialist should be early. However, we must consider that this delay may be because laryngeal symptoms may go unnoticed in the context of severe respiratory disease. Dyspnea or fatigue in a COVID-19 patient may be associated more with the pneumonia, interstitial fibrosis, polyneuropathy, or sarcopenia that patients suffer from than with possible laryngeal stenosis or vocal immobility if this possibility is not considered. Dysphonia may go unnoticed in critically ill patients after ICU discharge due to the presence of other more serious symptoms. The cases of dysphagia can be attributed to muscular debility after a long ICU stay. To avoid overlooking complications due to prolonged ETI, we consider it very important to request a post-extubation laryngeal examination for early diagnosis and treatment, or at least think about laryngeal causes if there is inspiratory obstruction.

The limitations of the study are several. First, despite being a multicenter study, nationally advertised, the size of the sample is low (*n* = 49). The main obstacle to patient recruitment has been the acceptance procedure of the different ethics committees of the collaborating hospitals, despite the high level of publicity due to SEORL. Moreover, having a control group with intubated patients without COVID-19 would have increased the value of our results, but it was not possible. Furthermore, data of endotracheal tube cuff pressures were not available in all patients.

More long-term studies are needed to observe complications, to evaluate sequelae, and to know which predictors are more relevant to avoid pharyngolaryngeal complications due to ETI in patients with COVID-19.

## 5. Conclusions

In COVID-19 infection, patients with comorbidities are at a higher risk of requiring prolonged mechanical ventilation. Postponing tracheostomy in prolonged ETI could influence the development of laryngeal lesions. A laryngeal examination may help to achieve an early diagnosis and treatment of immobility or laryngeal stenosis.

## Figures and Tables

**Table 1:** Participant hospitals.

Hospital Name	Community	Number of Cases
Hospital Universitario de Fuenlabrada	Community of Madrid	19
Hospital Universitario de Torrejón	Community of Madrid	7
Hospital de Sagunto	Community of Valencia	5
Hospital Universitario Parc Taulí Sabadell	Community of Catalonia	5
Hospital General de Valencia	Community of Valencia	4
Hospital Universitario Son Llàtzer	Community of Balearics Islands	3
Hospital Universitario Severo Ochoa	Community of Madrid	2
Hospital Universitario Galdakao Usansolo	Basque Country	2
Hospital Universitario Fundación Jimenez Diaz	Community of Madrid	2

**Table 2:** Baseline demographics.

**Demographics Characteristics**	
Gender (% male)	57.7%
Age (mean, range), years	60.6 (33–80)
**Comorbidities**	
None	8 (16.3%)
Obesity	23 (46.9%)
Arterial hypertension	21 (42.9%)
Dyslipidaemia	16 (32.7%)
Diabetes mellitus	14 (28.6%)
Respiratory insufficiency	8 (16.3%)
Hypothyroidism	4 (8.2%)
Tobacco use	13 (26.5%)

**Table 3:** General symptoms prior to intubation, and post-intubation ENT symptoms.

**General Symptoms Prior to Intubation**	
Dyspnea	34 (69.4%)
Fever	34 (69.4%)
Cough	33 (67.3%)
Asthenia	25 (51%)
Myalgia	20 (40.1%)
Headache	13 (26.5%)
Arthralgia	10 (20.4%)
Diarrhea	8 (16.3%)
Nausea/vomiting	5 (10.2%)
**Post-intubation ENT symptoms**	
Dysphonia	42 (87.8%)
Dysphagia	21 (42.9%)
Dyspnea	17 (34.7%)
Cough	8 (16.3%)
Throat clearing	10 (20.4%)
Stridor	5 (10.2%)

**Table 4:** Characteristics of intubation.

**Type of Intubation:**	
Orotracheal	48 (97.9%)
Nasotracheal	1 (2.2%)
**Intubations attempts** (mean, range)	1.3 (1–4)
**Pronation:**	
Number of patients	36 (73.5%)
Mean (range), days	5.3 (1–30)
**Tracheostomy:**	
Number of patients	22 (44.9%)
Mean of days of ETI previous tracheostomy	16.42 (7–31)
**Mean number of ETI days of extubation** (range), days	17.63 (4–77)

**Table 5:** Laryngoscopy findings.

**Location of Laryngoscopy Abnormality:**	
Glottis	44 (89.8%)
Subglottis	9 (18.4%)
Posterior glottis	53 (10.2%)
Arytenoids	3 (6.1%)
Tracheal	1 (2%)
Supraglottis	1 (2%)
**Type of laryngeal lesion:**	
Altered laryngeal mobility	39 (79.6%)
Subglottic stenoses	6 (12.2%)
Granulomas	6 (12.2%)
Atrophies	3 (6.1%)
Synechiae	2 (4%)
Tracheocutaneous fistula	1 (2%)
Glottic edema	1 (2%)

**Table 6:** Summary of reported laryngeal injuries after intubation for COVID-19.

AUTHOR	YEAR	NUMBER OF PATIENTS	SUBSITE	RELEVANT FINDINGS
Gervasio et al. [25]	2020	2	Subglottic	Stenoses
Naunheim et al. [10]	2020	20	Larynx	40% paralysis, 15% glottic stenoses, and 15% subglottic stenoses
Thong et al. [26]	2020	1	Glottic	Bilateral fixation of the cricoarytenoid joints and a granuloma
Fiacchini et al. [28]	2021	30	Larynx	33% tracheal lesions and 17% tracheoesophagic fistula
Leis-Cofiño et al. [12]	2021	79	Larynx	64% paralysis, 29% atrophy, and 7% granuloma
Neevel et al. [44]	2021	24	Larynx	Vocal fold motion impairment (50%), early glottic injury (39%), subglottic/tracheal stenosis (22%), and posterior glottic stenosis (17%)
Rouhani et al. [9]	2021	41		Alterations GRABS 54%
Sandu et al. [18]	2021	4	Subglottic	Stenoses
Scholfield et al. [11]	2021	3	Subglottic/ tracheal	Stenoses
Watson et al. [13]	2021	18	Larynx	Multiple injuries
Aibara et al. [41]	2022	40	Larynx	30% vocal fold immobility and 30% laryngeal granuloma
Allisan-Arrighi et al. [43]	2022	81	Glottic	Granulomas, arytenoid ankylosis, posterior/subglottic stenosis, and paralysis
Félix et al. [29]	2022	95	Larynx- tracheal	Stenoses and paralysis
Fiacchini et al. [38]	2022	16	Larynx	Glottic lesions and subglottic stenoses
Lechien et al. [2]	2022	188	Larynx	Dysphonia (with or without ETI)
Piazza et al. [24]	2022	14	Subglottic	Stenoses
Rapoport et al. [30]	2022	16	Larynx	No ETI, and paralysis
Sakihama et al. [42]	2022	6	Larynx	Posterior glottic synechiae/stenosis or subglottic/posterior glottic granulomas
Stratakos et al. [27]	2022	23	Larynx	Stenoses and tracheoesophageal fistulae

## Data Availability

Not applicable.

## Supplementary Materials

The following supporting information can be downloaded at: https://www.mdpi.com/article/10.3390/life13051207/s1; Video S1 (ankylosis), Video S2 (bilateral paralysis), and Video S3 (subglottic stenoses).
